# Distant Metastatic Pattern and Its Prognostic Significance in Malignant Pleural Mesothelioma: A Population‐Based Study Based on a Machine Learning Model

**DOI:** 10.1111/crj.70133

**Published:** 2025-11-11

**Authors:** Jian Yu, Chi Peng, Qianwen Ye, Cong Huo, Binrui Gao, Qing Wei, Yibo Li, Kaidi Yang

**Affiliations:** ^1^ Department of Oncology Hainan Hospital of Chinese People's Liberation Army General Hospital Sanya Hainan Province China; ^2^ Department of Health Statistics Naval Medical University Shanghai China; ^3^ Department of Respiratory Hainan Hospital of Chinese People's Liberation Army General Hospital Sanya Hainan Province China; ^4^ Department of Orthopedics Central war Zone General Hospital Wuhan Hubei China

**Keywords:** distant metastasis, malignant pleural mesothelioma, overall survival, prognostic model, retrospective study

## Abstract

**Background:**

Malignant pleural mesothelioma (MPM) is an insidious and aggressive tumor, often hindering timely clinical interventions. Despite its clinical relevance, epidemiological research focusing on MPM metastases remains limited.

**Methods:**

We conducted a retrospective review of MPM cases with site‐specific metastasis records from the Surveillance, Epidemiology, and End Results (SEER) between 2010 and 2019. Propensity Score Matching was employed to minimize bias between distant metastasis and non‐distant metastasis groups. A prognostic model for predicting overall survival was established using clinical variables derived from Lasso regression. Variable importance for survival outcomes was estimated using the Random Survival Forests algorithm. The performance of the nomogram was evaluated using the receiver operating characteristic (ROC) curves and calibration plots.

**Results:**

The presence of distant metastasis significantly reduced median overall survival from 10.5 to 7 months, with further detriment observed in cases with sarcomatoid histology and without chemotherapy intervention. Multivariable analysis identified sarcomatoid subtype, T4 stage, N1+ nodal involvement, and bilateral disease as significant predictors of increased metastatic potential. Histology, surgery, and metastasis status emerged as the top three clinical variables influencing survival. The nomogram demonstrated strong discrimination and calibration for predicting the 1‐year and 3‐year overall survival in both training and validation cohorts. The contralateral lung was the most frequent site of distant metastasis, with lymph node metastasis presenting a significantly better prognosis than that observed in patients with metastases to other organs.

**Conclusions:**

The large population‐based analysis provides a comprehensive characterization of site‐specific metastases in MPM. The identified risk factors can help stratify patients at higher risk for metastatic progression and support early, targeted clinical decision‐making.

## Graphical abstract

The presence of distant metastasis significantly reduced the median overall survival in patients with malignant pleural mesothelioma (MPM). Histologic subtype, T‐stage, N‐stage, and tumor laterality are independent risk factors for metastatic spread. Multivariable analysis highlights sarcomatoid histology, T4 stage, N1+ nodal involvement, and bilateral disease as key predictors of increased metastatic potential. The contralateral lung is the most frequent metastatic site, while solitary lymph node metastasis is associated with a comparatively favorable prognosis.

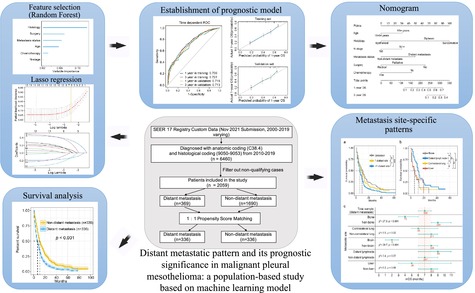

## 1 Introduction

Malignant pleural mesothelioma (MPM) is a rare, aggressive, and currently incurable malignancy originating from mesothelial cells in the lining layers of the pleural cavity [1]. Its occurrence is closely linked to occupational and environmental asbestos exposure [2]. The period between asbestos exposure and symptomatic diagnosis ranges from 14 to 72 years, which means many people are diagnosed at advanced stages [3]. Although MPM is typically characterized by local infiltration into intrathoracic structures such as the ribs, spine, mediastinum, and contralateral pleura, its insidious growth and nonspecific symptoms often lead to metastasis upon diagnosis [4].

Previous reports have documented metastatic spread of MPM to various distant organs, including liver [5], brain [6], bone [7], and skin [8]. However, only 13.8%–23% of MPM cases are diagnosed at a stage amenable to surgical resection [9, 10, 11], underscoring the critical need for a better understanding of the patterns, determinants, and prognostic impact of distant metastases in this disease. While distant metastases are major contributors to cancer‐related mortality in many malignancies [12], their role in MPM prognosis remains poorly defined. Also, the extent to which the dismal prognosis of MPM is attributed to distant metastasis remains unclear. Existing studies on organ‐specific metastatic impact in MPM are sparse, emphasizing the need for a comprehensive, population‐based investigation.

Therefore, the primary objective of our study is to explore the relevance of distant metastases, along with site‐specific patterns, on the overall survival of MPM using the Surveillance, Epidemiology, and End Results (SEER) database. We aim to identify clinical variables influencing both patient survival and distant metastasis status, and to develop a clinically applicable prognostic model. This large‐scale, population‐based analysis can deepen our understanding of how clinical variables impact distant metastases in MPM, thereby informing more effective and individualized treatment strategies.

## 2 Methods

### 2.1 Data Source

**FIGURE 1:**
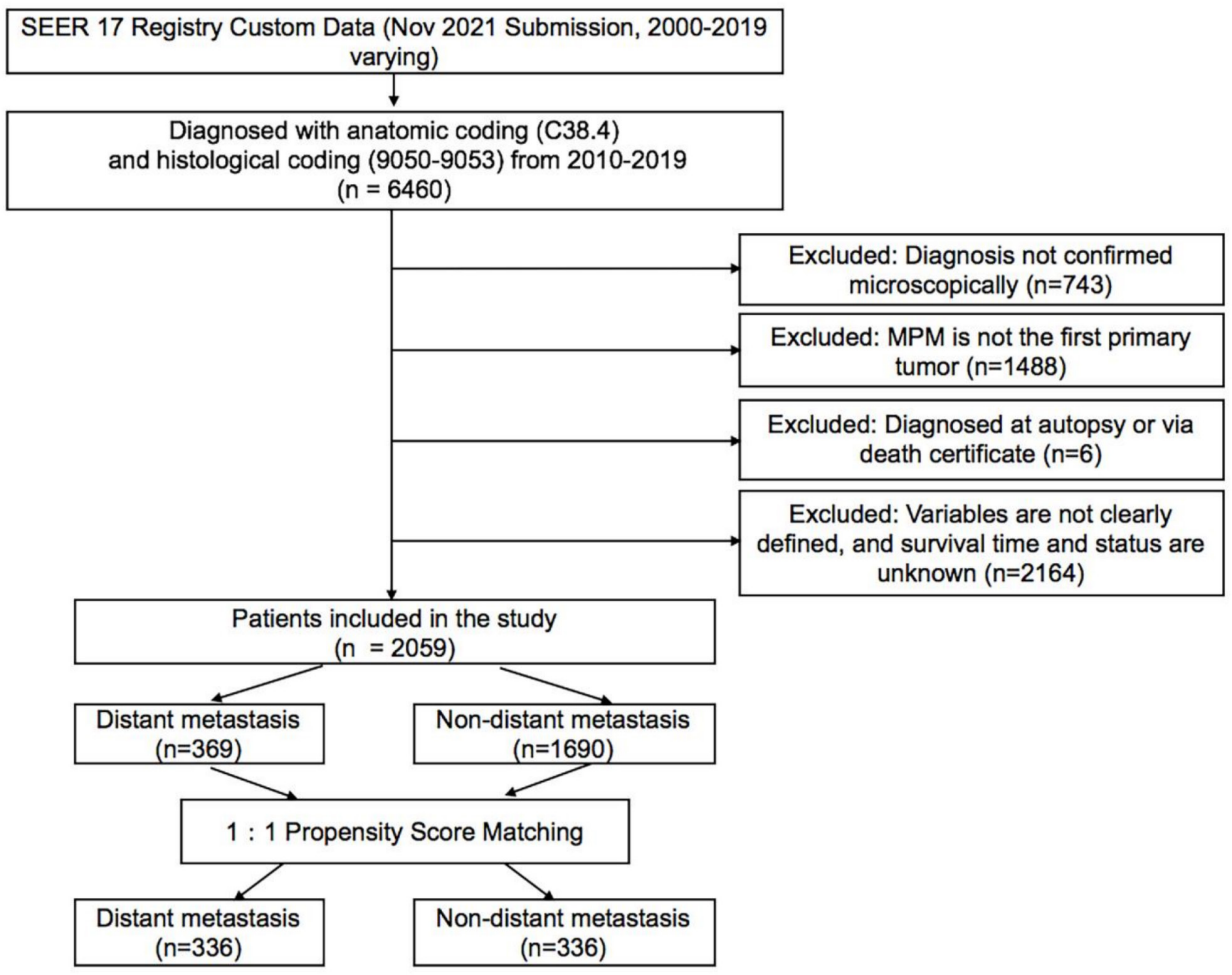
The flow chart showing the selection of eligible patients for the following analysis. MPM, malignant pleural mesothelioma.

The SEER database https://seer.cancer.gov, which captures approximately 30% of incident cancer cases in the United States, was used as the data source for this study [13]. Because the database contains only deidentified patient information, written informed consent or institutional review board approval was not required. Data were extracted from the SEER 17 Registry Custom Data (November 2021 Submission, 2000–2019) using the software SEER*Stat (Version 8.4.0) under access ID 10160‐Nov2021. MPM cases were identified using the International Classification of Diseases for Oncology, 3rd Edition (ICD‐O‐3/WHO 2008), based on anatomic site code C38.4 and histological code 9050‐9053. Given the absence of detailed metastasis information prior to 2010, our analysis only focused on patients diagnosed after this period. To ensure data reliability, we limited the cohort to patients with microscopically confirmed MPM as their first primary tumor. Cases identified only from autopsy or death certificate were excluded. Additionally, patients with missing clinical variable data were excluded to reduce potential confounding. The inclusion and exclusion criteria are summarized in Figure 1.

### 2.2 Definition of Clinical Variables

The following clinical and demographic variables were extracted: age, sex, race, marital status, histology, laterality, Tumor‐stage (T‐stage), Lymph node‐stage (N‐stage), metastasis status, median household income, treatment modalities, and survival outcomes. These were categorized as follows: (i) age: 18–64/> 65 years; (ii) sex: female/male; (iii) race: White/Black/Other (including Asian, Pacific Islander, American Indian, and Alaska Native); (iv) marital status: married/never married/other; (v) histology: biphasic/epithelioid/sarcomatoid; (vi) laterality: bilateral/left/right; (vii) T‐stage: T1/T2/T3/T4 (AJCC 7th Edition); (viii) N‐stage: N0/N1+ (where N1+ includes N1, N2, and N3; AJCC 7th Edition); (ix) surgery: no surgery/local excision/radical surgery; (x) chemotherapy: yes/no; (xi) radiotherapy: yes/no; (xii) median household income: >$60,000/≤$60,000; (xiii) metastasis status: M0 (non‐metastatic)/M1 (metastatic); (xiv) Overall survival: measured in months from diagnosis to death or last follow‐up. Moreover, specific sites of distant metastasis were recorded, including distant lymph nodes, bone, contralateral lung, liver, brain, and a category labeled “Not Otherwise Specified” (NOS). A site‐specific summary of metastasis patterns is provided in Table S1.

### 2.3 Propensity Score Matching (PSM)

To mitigate confounding effects between the metastatic (M1) and nonmetastatic (M0) groups, we employed PSM, an analytic approach based on a logistic regression model that accounts for the independent correlations of covariables (i–xi) with metastasis status (xii) [14]. Patients were matched in a 1:1 ratio to their nearest neighbor without replacement, ensuring comparability by balancing observed covariates. A standardized mean difference of less than 0.10 was used as the threshold to indicate adequate balance. The matching procedure were executed using Stata software (v16.0).

### 2.4 Prognostic Model Construction

To avoid model overfitting, we applied Lasso‐penalized Cox regression using the *glmnet* R package. A 10‐fold cross‐validation was used to determine the optimal penalty parameter (*λ*), with lambda.1se (the largest λ within one standard error of the minimum) selected for variable inclusion. Clinical variables with nonzero coefficients at this λ value were incorporated into the final model. Variable importance was estimated using the Random Survival Forest algorithm incorporated into the *randomForestSRC* R package. Prognostic accuracy was evaluated through time‐dependent receiver operating characteristic (ROC) curve analysis using the *survivalROC* package. For multivariable survival analysis, a Cox proportional hazards model was constructed with the selected variables using the *rms* package, and a nomogram was then established to predict the probability of 1‐year and 3‐year overall survival rates in MPM patients. A calibration curve was used to assess the nomogram's calibration ability by comparing actual prognosis with predicted survival. The discrimination ability of the nomogram was evaluated using the concordance index (C‐index).

### 2.5 Statistical Analysis

Overall survival was estimated using Kaplan–Meier analysis with the *survival* and *survminer* R packages. Group comparisons were performed using the log‐rank test. A forest plot was generated to illustrate survival differences between metastatic and non‐metastatic groups, including 95% confidence intervals. Univariable and multivariable logistic regression analyses were conducted to identify factors associated with distant metastasis. All visualizations were created using the *ggplot2* package. Statistical analyses were conducted using RStudio (version 4.2.0), and a two‐sided *p* < 0.05 was considered statistically significant.

## 3 Results

### 3.1 Selection of MPM Cohort From SEER for PSM

From the SEER database, a total of 2059 MPM patients with complete clinical records were extracted, of which 369 had confirmed distant metastases. Initial comparisons between metastatic and non‐metastatic groups revealed notable imbalances in key clinical variables, particularly N‐stage, T‐stage, and surgical status, with standardized biases exceeding 10%. To reduce confounding and improve comparability, 1:1 PSM was performed, pairing patients in the metastatic group with those in the non‐metastatic group (Figure S1), as shown in Table 1, where no significant differences were observed in demographic and clinical variables post‐matching, indicating effective bias control.

**TABLE 1:** Baseline characteristics before and after propensity score matching (PSM), showing statistical comparisons between distant metastasis (central group for comparison, highlighted in gray) and non‐distant metastasis groups (*χ* ^2^ test).

Factor	Pre‐PSM		Distant metastasis (*n* = 336)	Post‐PSM	
	Non‐distant metastasis all (*n* = 1690)	Comparison (*p*‐value)		Non‐distant metastasis (*n* = 336)	Comparison (*p*‐value)
**Age (*n*, %)**		***χ*** ^**2**^ **= 4.02, 0.08**			***χ*** ^**2**^ **= 0.53, 0.47**
18–64 years	383 (22.7%)		83 (24.7%)	74 (22%)	
65 + years	1307 (77.3%)		253 (75.3%)	262 (78%)	
**Sex (*n*, %)**		***χ*** ^**2**^ **= 0.24, 0.62**			***χ*** ^**2**^ **= 0.01, 0.93**
Female	371 (22%)		76 (22.6%)	74 (22%)	
Male	1319 (78%)		260 (77.4%)	262 (78%)	
**Race (*n*, %)**		***χ*** ^**2**^ **= 7.81, 0.02**			***χ*** ^**2**^ **= 0.39, 0.82**
Black	77 (4.6%)		20 (6%)	17 (5.1%)	
Other	79 (4.7%)		21 (6.2%)	19 (5.7%)	
White	1534 (90.8%)		295 (87.8%)	300 (89.3%)	
**Marital status (*n*, %)**		***χ*** ^**2**^ **= 0.52, 0.77**			***χ*** ^**2**^ **= 0.31, 0.86**
Married	1150 (68%)		233 (69.3%)	236 (70.2%)	
Never married	154 (9.1%)		30 (8.9%)	26 (7.7%)	
Other	386 (22.8%)		73 (21.7%)	74 (22%)	
**Histology (*n*, %)**		***χ*** ^**2**^ **= 9.33, 0.01**			***χ*** ^**2**^ **= 2.13, 0.35**
Biphasic	285 (16.9%)		39 (11.6%)	34 (10.1%)	
Epithelioid	1124 (66.5%)		230 (68.5%)	247 (73.5%)	
Sarcomatoid	281 (16.6%)		67 (19.9%)	55 (16.4%)	
**Laterality (*n*, %)**		***χ*** ^**2**^ **= 46.92, < 0.001**			***χ*** ^**2**^ **= 0.70, 0.70**
Bilateral	7 (0.4%)		4 (1.2%)	2 (0.6%)	
Left	657 (38.9%)		137 (40.8%)	140 (41.7%)	
Right	1026 (60.7%)		195 (58%)	194 (57.7%)	
**T stage (*n*, %)**		***χ*** ^**2**^ **= 81.57, < 0.001**			***χ*** ^**2**^ **= 0.16, 0.98**
T1	544 (32.2%)		91 (27.1%)	95 (28.3%)	
T2	388 (23%)		60 (17.9%)	58 (17.3%)	
T3	372 (22%)		46 (13.7%)	44 (13.1%)	
T4	386 (22.8%)		139 (41.4%)	139 (41.4%)	
**N stage (*n*, %)**		***χ*** ^**2**^ **= 40.40, < 0.001**			***χ*** ^**2**^ **= 0.60, 0.44**
N0	1137 (67.3%)		174 (51.8%)	185 (55.1%)	
N1+	553 (32.7%)		162 (48.2%)	151 (44.9%)	
**Surgery (*n*, %)**		***χ*** ^**2**^ **= 57.46, < 0.001**			***χ*** ^**2**^ **= 1.73, 0.42**
No surgery	982 (58.1%)		259 (77.1%)	270 (80.4%)	
Local excision	511 (30.2%)		60 (17.9%)	55 (16.4%)	
Radical surgery	197 (11.7%)		17 (5.1%)	11 (3.3%)	
**Chemotherapy (*n*, %)**		***χ*** ^**2**^ **= 1.05, 0.30**			***χ*** ^**2**^ **= 0.06, 0.81**
No	614 (36.3%)		118 (35.1%)	114 (33.9%)	
Yes	1076 (63.7%)		218 (64.9%)	222 (66.1%)	
**Radiotherapy (*n*, %)**		***χ*** ^**2**^ **= 0.70, 0.40**			***χ*** ^**2**^ **= 0.11, 0.75**
No	1405 (83.1%)		284 (84.5%)	288 (85.7%)	
Yes	285 (16.9%)		52 (15.5%)	48 (14.3%)	
**Income (*n*, %)**		***χ*** ^**2**^ **= 1.68, 0.19**			***χ*** ^**2**^ **= 0.07, 0.78**
$60 000+	1219 (72.1%)		251 (74.7%)	255 (75.9%)	
< $60 000	471 (27.9%)		85 (25.3%)	81 (24.1%)	

Abbreviations: CI: confidence interval; HR: hazard ratio; Income: median household income; N‐stage: lymph node stage; T‐stage: tumor stage.

### 3.2 Survival Analysis After PSM

**FIGURE 2:**
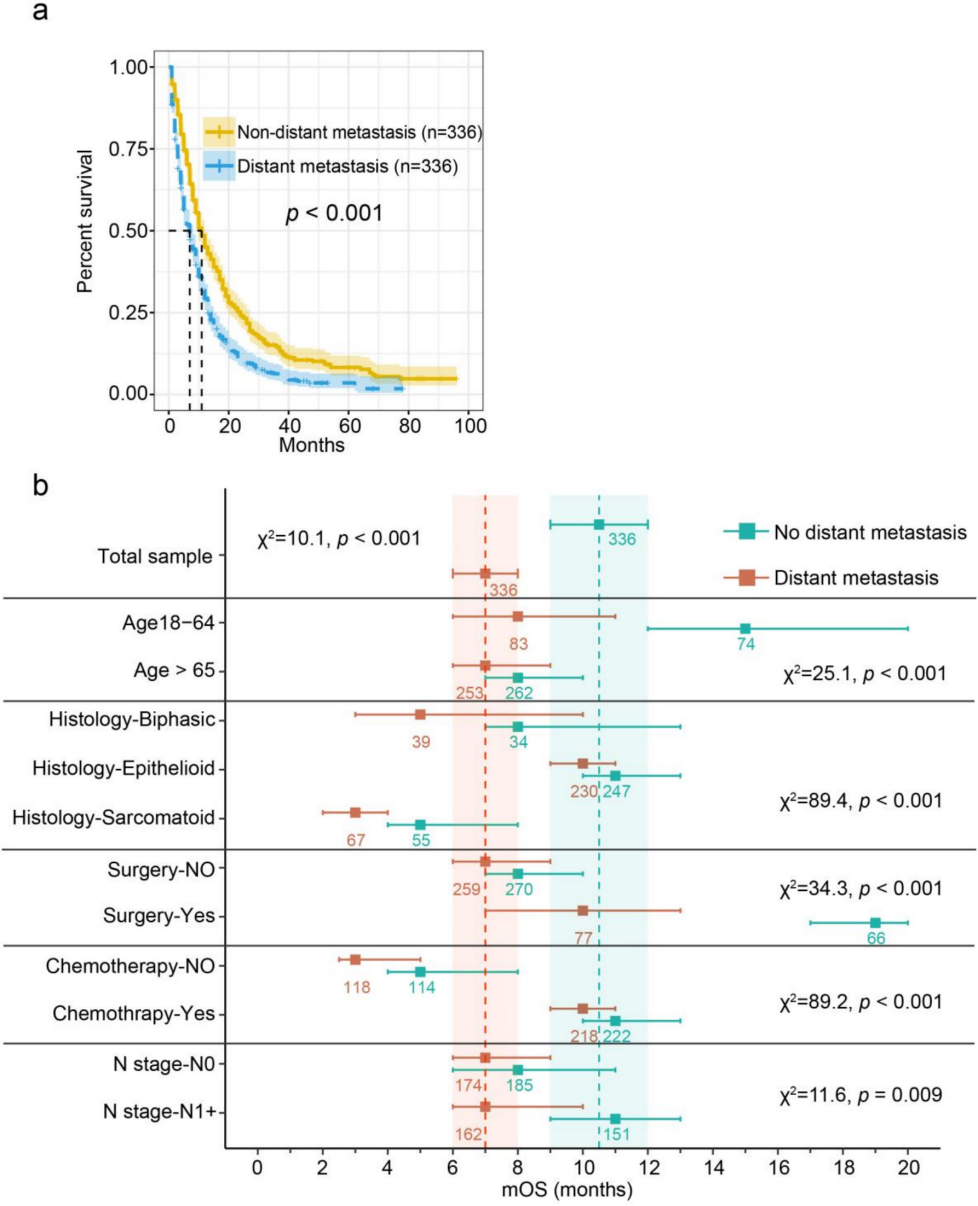
(a) Kaplan–Meier survival curves for MPM patients with and without distant metastasis from the total propensity score matching (PSM) cohort. (b) The forest plot illustrates the median overall survival (mOS, months) ± 95% confidence intervals for MPM patients stratified by distant metastasis status and key prognostic factors. Numbers indicate sample sizes for each subgroup. Dashed lines and shaded areas cover the median and ± 95% confidence intervals for the total MPM samples upon PSM. Results of log‐rank tests are shown for each group.

Following PSM, overall survival was compared between the matched groups (*n* = 336 per group). Patients with distant metastasis exhibited a significantly shorter median overall survival (mOS: 7 months) compared to those in the non‐distant metastasis group (mOS: 10.5 months; log‐rank test: *p* < 0.001; see Figure 2a).

### 3.3 Identifying the Clinical Risk Factors Associated With Distant Metastases of MPM

To identify risk factors associated with distant metastasis in MPM, we conducted logistic regression analyses. Histology, T‐stage, N‐stage, and laterality were significantly associated with distant metastases in both univariable and multivariable models. Of note, the sarcomatoid subtype, T4, N1+, and bilateral MPM were associated with increased metastatic potential and confirmed as independent risk factors (Table 2).

**TABLE 2:** Univariable and multivariable logistic regression analyses of clinical risk factors associated with distant metastases in MPM patients.

Factor	Univariate analysis		Multivariate analysis	
	OR (95% CI)	*p‐*value	OR (95% CI)	*p*‐value
**Age**				
18–64 years	Ref		Ref	
65 years	0.82 (0.63–1.06)	0.136	0.87 (0.66–1.16)	0.348
**Sex**				
Female	Ref			
Male	0.93 (0.71–1.21)	0.571	0.91 (0.68–1.21)	0.521
**Race**				
Black	Ref			
Other	1.28 (0.68–2.43)	0.44	1.48 (0.75–2.94)	0.258
White	0.73 (0.44–1.18)	0.199	0.78 (0.46–1.31)	0.344
**Marital status**				
Married	Ref			
Never married	1.10 (0.75–1.62)	0.625	1.03 (0.68–1.57)	0.888
Other	1.09 (0.83–1.42)	0.544	1.14 (0.85–1.52)	0.381
**Histology**				
Biphasic	Ref		Ref	
Epithelioid	1.02 (1.13–2.31)	0.008	1.01 (1.12–2.34)	0.11
Sarcomatoid	1.88 (1.23–2.85)	0.003	1.92 (1.24–2.98)	0.004
**Laterality**				
Bilateral	Ref			
Left	0.09 (0.04–0.22)	< 0.001	0.13 (0.05–0.32)	< 0.001
Right	0.08 (0.03–0.20)	< 0.001	0.10 (0.04–0.26)	< 0.001
**T stage**				
T1	Ref			
T2	0.93 (0.66–1.32)	0.703	0.82 (0.57–1.17)	0.267
T3	0.72 (0.50–1.05)	0.092	0.56 (0.38–0.83)	0.004
T4	2.55 (1.91–3.39)	< 0.001	2.05 (1.52–2.77)	< 0.001
**N stage**				
N0	Ref		Ref	
N1+	2.09 (1.66–2.62)	< 0.001	2.12 (1.66–2.71)	< 0.001
**Income**				
> $60 000 0–$60 000	Ref 0.83 (0.64–1.08)	0.174	0.87 (0.66–1.15)	0.333

Abbreviations: HR: hazard ratio; Income: median household income; N‐stage: lymph node stage; NCI: confidence interval; T‐stage: tumor stage.

### 3.4 Establishment of a Prognostic Model Using Clinical Variables

**FIGURE 3:**
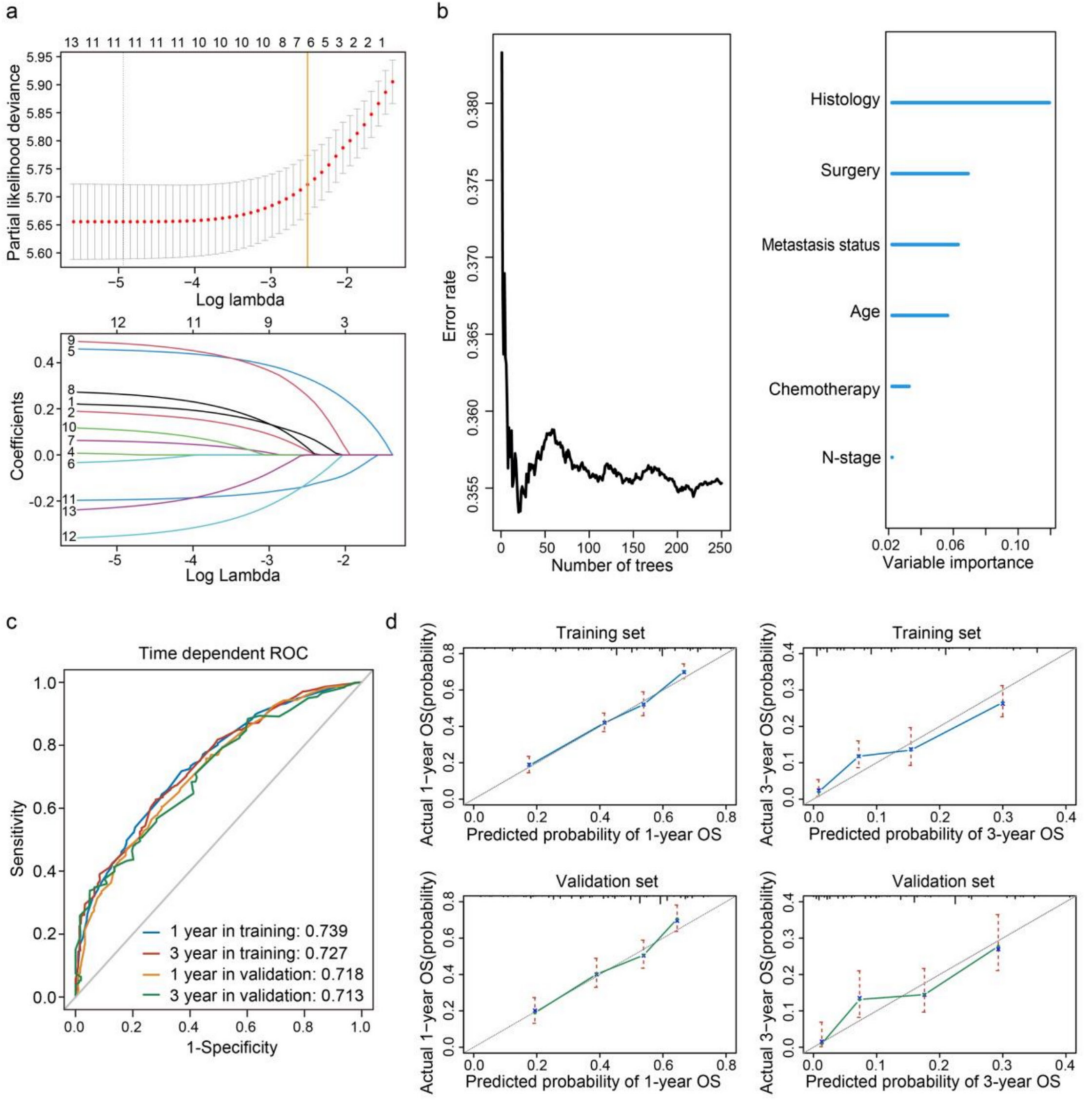
(a) The panel below illustrates the coefficient paths of thirteen clinical variables against the penalty parameter (lambda). As lambda increases, more coefficients are shrunk to zero. The optimal lambda (lambda.1se) was selected via 10‐fold cross‐validation (vertical orange line), yielding the most regularized model within one standard error of the minimum error. At this value, six variables retained non‐zero coefficients. (b) Error rates according to the number of trees generated in the simplified Random Forest Survival model (left panel). The right panel shows the ordered variable importance of distant metastasis, histology, surgery, age, chemotherapy, and N‐stage. (c) The receiver operating characteristics (ROC) curves depicted the 1‐year and 3‐year area under the ROC curve (AUC) in the training and validation cohorts. (d) The calibration curves for the nomogram for predicting 1‐year and 3‐year overall survival (OS) in the MPM training and validation set (bootstrap 1000 repetitions). The x‐axis represents the predicted probability of overall survival. The y‐axis represents the actual probability.

We next sought to develop a predictive model for overall survival using selected clinical variables. To optimize the predictive model and prevent overfitting, a 10‐fold cross‐validation Lasso regression process was employed, resulting in the screening of six variables (histology, surgery, metastasis status, age, chemotherapy, and N‐stage) from the initial model (Figure 3a). Among these factors, surgical treatment substantially improved survival in non‐metastatic patients (HR [95% CI]: 0.47 [0.35–0.64], *p* < 0.001). Meanwhile, outcomes were deteriorated by sarcomatoid histology (vs. epithelioid: HR 2.78 [2.08–3.73], *p* < 0.001; vs. biphasic: 1.48 [1.18–1.78], *p* < 0.001) and absence of chemotherapy (2.52 [1.96–3.24], *p* < 0.001) for the metastasis group (Figure 2b, Figure S2).

To evaluate the relative contribution of each variable to patient survival, we applied the Random Survival Forest algorithm, a machine‐learning method designed for time‐to‐event survival data [15]. Among all features, histology emerged as the most important prognostic factor (importance score: 0.127), aligning with previous findings [16]. Metastasis status ranked third (importance score: 0.057), following surgery (0.066), underscoring its substantial impact on survival (Figure 3b). Its prognostic relevance was further confirmed in the multivariable Cox regression analysis, where metastasis status remained an independent predictor of overall survival (Table 3). Subgroup analyses stratified by histologic subtypes confirmed the consistent negative impact of metastasis status, while chemotherapy demonstrated significant survival benefits across all histologic categories. In contrast, the effect of surgical intervention appeared to be subtype‐specific: sarcomatoid subtype appears to derive greater benefit from local excision, whereas radical surgery was relatively more advantageous in biphasic patients (Figure S3).

**TABLE 3:** Univariable and multivariable Cox regression analyses of predictive variables correlated with distant metastasis in MPMs. Of note, meaningful variables through lasso‐regression screening were incorporated for multivariable analysis.

Factor	Univariate analysis		Multivariate analysis	
	HR (95% CI)	*p*‐value	HR (95% CI)	*p*‐value
**Age**				
18–64 years	Ref	< 0.001	Ref	< 0.05
> 65 years	1.47 [1.31–1.65]		1.35 [1.17–1.56]	
**Sex**				
Female	Ref	< 0.001		
Male	1.41 [1.25–1.59]			
**Race**				
Black	Ref	0.593		
Other	1.04 [0.77–1.42]			
White	0.94 [0.75–1.18]			
**Marital status**				
Married	Ref	0.652		
Never married	1.06 [0.90–1.26]			
Other	1.04 [0.93–1.17]			
**Histology**				
Epithelioid	Ref	< 0.001	Ref	< 0.001
Biphasic	1.73 [1.48–2.02]		1.96 [1.67–2.27]	
Sarcomatoid	2.69 [2.29–3.11]		2.54 [2.17–2.98]	
**Laterality**				
Bilateral	Ref	0.294		
Left	1.25 [0.80–1.95]			
Right	1.31 [0.84–2.05]			
**T stage**				
T1	Ref	< 0.001		
T2	0.94 [0.82–1.07]			
T3	0.82 [0.72–0.94]			
T4	1.29 [1.14–1.47]			
**N stage**				
N0	Ref	< 0.05	Ref	< 0.05
N1+	1.18 [1.07–1.31]		1.34 [1.19–1.51]	
**Distant metastasis**				
No	Ref	< 0.001	Ref	< 0.001
Yes	1.67 [1.48–1.88]		1.61 [1.38–1.89]	
**Surgery**				
No surgery	Ref	< 0.001	Ref	< 0.001
Local excision	0.63 [0.56–0.70]		0.67 [0.58–0.76]	
Radical surgery	0.51 [0.43–0.6]		0.53 [0.43–0.66]	
**Chemotherapy**				
No	Ref	< 0.001	Ref	< 0.001
Yes	0.69 [0.62–0.76]		0.64 [0.57–0.73]	
**Radiotherapy**				
No	Ref	< 0.001		
Yes	0.72 [0.63–0.82]			
**Income**				
> $60 000 0–$60 000	Ref 1.15 [1.04–1.28]	< 0.05		

Abbreviations: CI: confidence interval; HR: hazard ratio; Income: median household income; N‐stage: lymph node stage; T‐stage: tumor stage.

**FIGURE 4:**
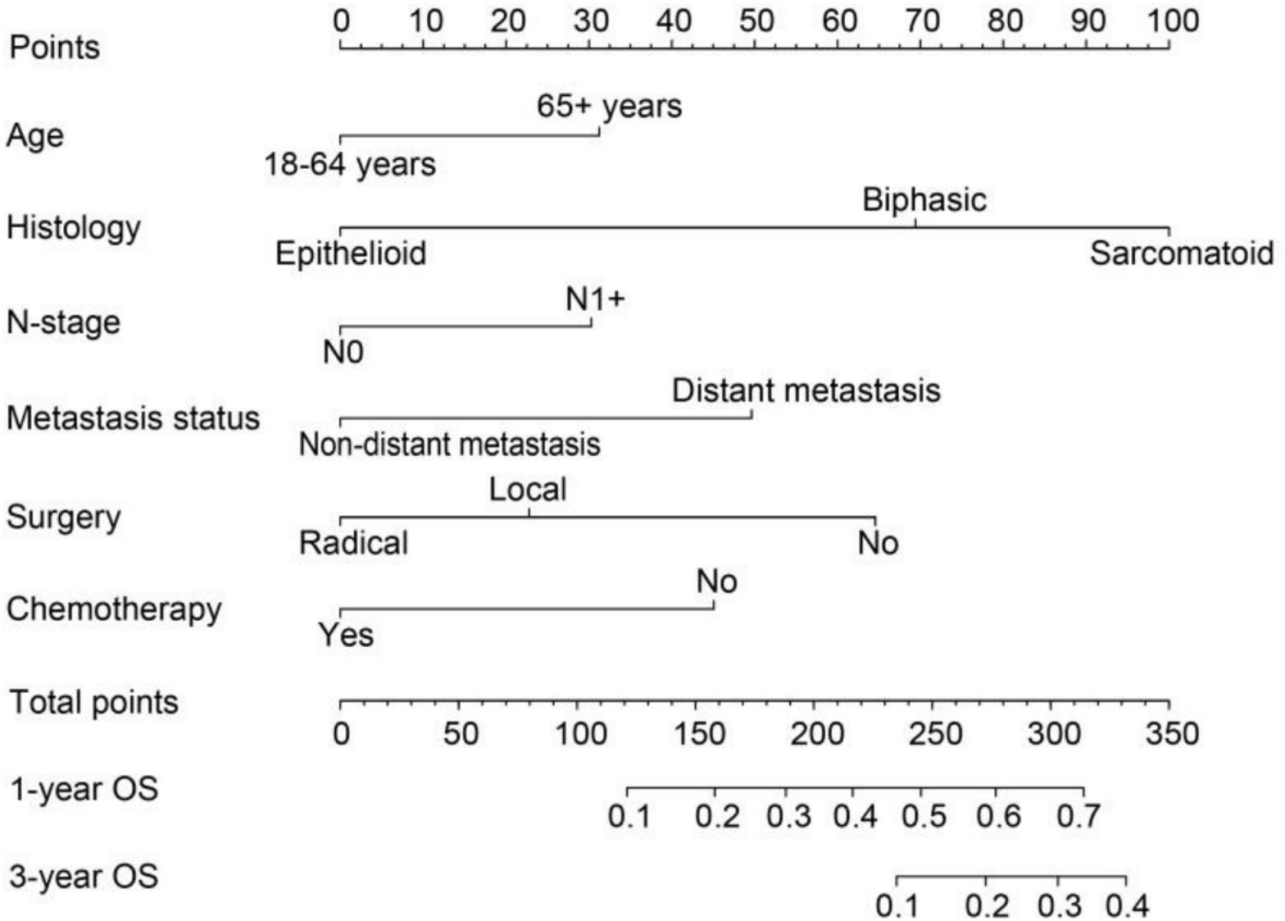
Nomogram for predicting 1‐year and 3‐year overall survival in patients with MPMs. The total scores calculated from six clinical variables corresponding to a predicted probability of survival.

Following random assignment of all MPM patients into a training set and a test set at a 7:3 ratio, we assessed the predictive performance of the survival model using ROC curve analysis. The model demonstrated favorable discriminatory ability, with 1‐ and 3‐year area under the curve (AUC) values of 0.739 and 0.727 in the training set, and 0.718 and 0.713 in the validation set, respectively (Figure 3c). A nomogram was subsequently developed based on the multivariable Cox regression results to estimate 1‐ and 3‐year overall survival probabilities (Figure 4). The C‐index value for the model reached 0.698 (95% CI: 0.682–0.713), indicating good predictive accuracy. Calibration curves further demonstrated strong agreement between the predicted and observed survival outcomes for 1‐ and 3‐year in both the training and validation cohorts, supporting the robustness and reliability of the model (Figure 3d).

### 3.5 Comparison of Patient Survival by Solitary Location and Number of Distant Metastatic Lesions

**FIGURE 5:**
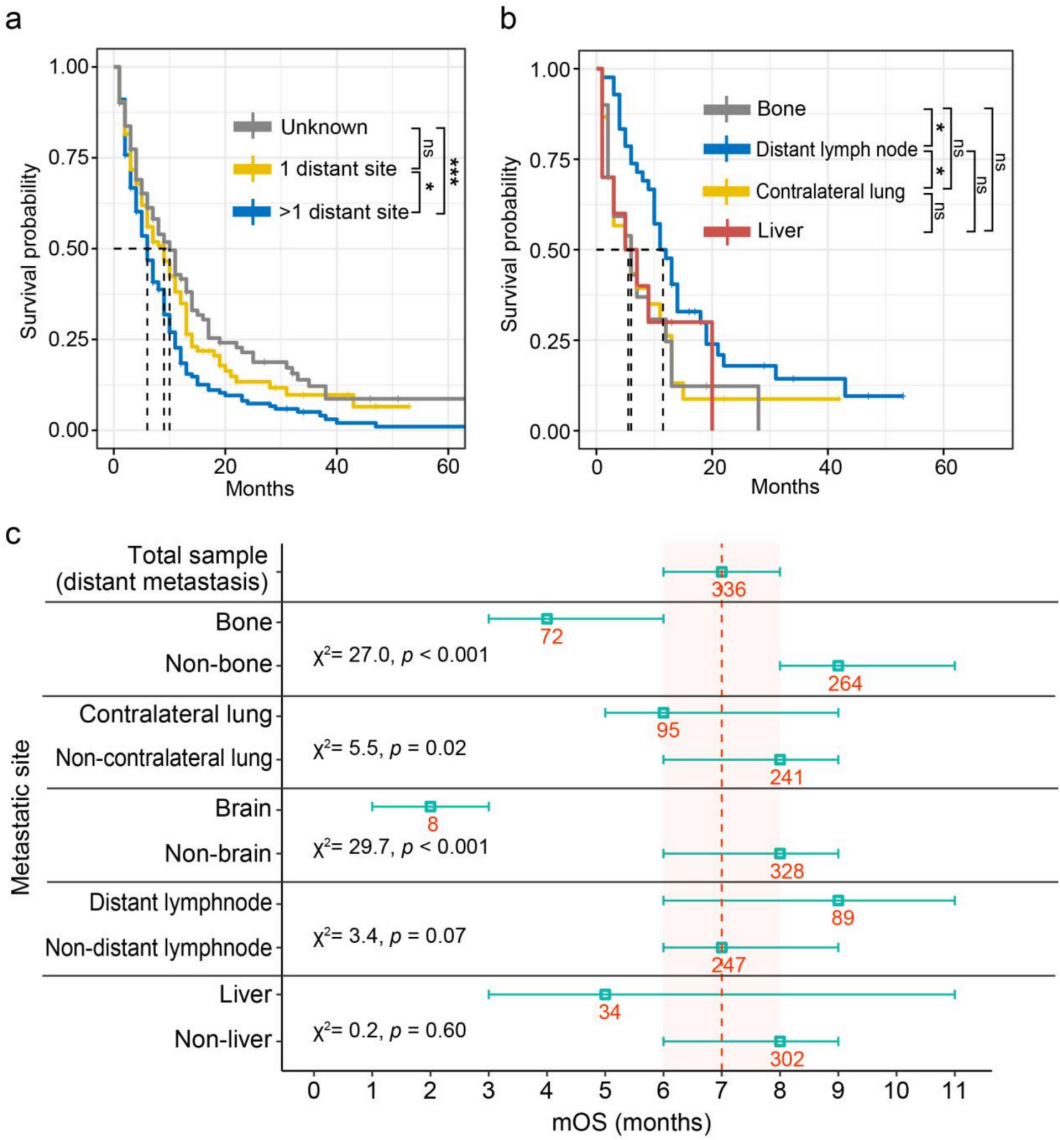
Kaplan–Meier survival curves of overall survival stratified by (a) the number of distant metastatic lesions and (b) the site of solitary metastasis. ***p < 0.01; **p < 0.01; *p < 0.05; ns, not significant. (c) The forest plot illustrates the mOS (months) ± 95% confidence intervals for patients with and without metastasis at specific sites, including bone, lung, brain, distant lymph node, and liver. Numbers indicate sample sizes for each subgroup. Dashed lines and shaded areas cover the median and ± 95% confidence intervals of corresponding groups. Results of log‐rank tests are shown for each group.

Different metastatic sites are associated with varying impacts on overall survival in cancer patients [17, 18]. For MPM, the most frequently involved metastatic sites were the contralateral lung (95, 18.96%), followed by distant lymph nodes (89, 17.76%), bone (72, 14.40%), liver (34, 6.78%), brain (8, 1.60%), and sites not otherwise specified (NOS). Stratifying by the number of metastatic lesions revealed that patients harboring multiple distant lesions (mOS: 6 months) had a significantly worse prognosis than those with a single metastatic lesion (Figure 5a; mOS: 9 months, log‐rank test: *p* < 0.001).

Further analysis by solitary metastatic site indicated that patients with isolated distant lymph node involvement alone exhibited the most favorable prognosis (Figure 5b). It aligns with previous findings that lymphatic colonization may precede involvement of remote organs [19]. Due to the limited number of patients with solitary brain metastases, we assessed site‐specific survival regardless of whether metastases were solitary or multiple. As shown in Figure 5c, involvement of the bone, brain, and contralateral lung was significantly associated with poorer survival. In contrast, patients with distant lymph node metastases exhibited relatively better survival outcomes compared to those with other metastatic sites.

## 4 Discussion

MPM often develops covertly within the thoracic cavity, typically reaching an advanced stage by the time clinical symptoms prompt medical evaluation. While local invasion is a well‐recognized feature, distant metastasis has historically been documented primarily through isolated case reports with biopsy confirmation [20, 21]. Most large‐scale analyses of metastatic spread have relied on postmortem examinations [22, 23]. For instance, Falconieri et al. reviewed 171 autopsied MPM cases and reported that 54% exhibited distant metastases [22]. Even more striking, Rhian S. Finn et al. reported the existence of extra‐pleural dissemination in 87.7% of cases, highlighting the extent to which such dissemination may be underrecognized during life [23]. Traditionally, distant metastases in MPM were considered to have limited clinical significance [2], with the prevailing belief that most patients succumb to complications arising from local tumor progression rather than systemic dissemination [24]. However, this view has been challenged by recent findings, including those of Yamagishi et al. [25], who demonstrated that brain metastases are associated with significantly worse prognoses in MPM patients. To reconcile these contradictory views and comprehensively evaluate the incidence, prognostic implications, and risk factors of distant metastasis in MPM, we performed a pooled analysis using data from the SEER database. This analysis aims to provide new insights that can support improved clinical decision‐making in managing MPM patients.

As demonstrated in our findings, histologic subtype emerges as the most influential factor affecting patient survival in MPM, with nonepithelioid histologic subtypes serving as negative prognostic indicators. By contrast, MPMs with an epithelioid histologic type tend to exhibit slow and localized growth, leading to relatively favorable outcomes. Notably, histologic classification is not only prognostically informative but also instrumental in guiding therapeutic strategies. For instance, surgery is predominantly recommended for epithelioid MPM, emphasizing the tailored approach based on histology [26]. It has previously been reported that distant tumor metastases are typically associated with the sarcomatous component of biphasic MPMs [27], suggesting that sarcomatoid cells acquire distinct biological capabilities compared to their epithelioid counterparts. Emerging evidence implicates epithelial‐mesenchymal transition (EMT) signaling in driving invasiveness and progression in mesothelioma and other cancers. Sarcomatoid components have been strongly linked to EMT activation, TP53 signaling, dysregulated cell cycling, angiogenesis, and upregulation of immune checkpoint pathways [28]. These associations imply potential regulatory mechanisms underlying the evident distant metastasis observed in sarcomatous MPMs.

Beyond histological classification, we identified surgery as the most critical factor influencing mesothelioma prognosis. However, the selection of the optimal surgical approach remains controversial, and determining how to choose the best procedure based on individual patient characteristics has become an active area of research. Our analysis demonstrates that both limited and radical surgery are associated with improved survival in MPM patients. Notably, the sarcomatoid subtype appeared to derive greater benefit from limited surgery, whereas radical surgery showed relatively greater advantage in patients with the biphasic subtype. Both procedures were performed more frequently in non‐metastatic patients (41.9%) than in their metastatic counterparts (19.7%). Although a lower hazard ratio (HR) was observed for radical surgery, limited surgery was statistically noninferior (*p* > 0.05). The wider confidence intervals associated with radical surgery suggest considerable heterogeneity in treatment outcomes within this patient group. This finding aligns with existing literature indicating that radical procedures such as extrapleural pneumonectomy (EPP) provide no definitive survival advantage over non‐EPP approaches [29]. Both our data and recent clinical developments support the ongoing trend of EPP being progressively phased out in favor of conservative surgical strategies, such as Pleurectomy/Decortication (P/D) and extended pleurectomy (E/P) [30, 31].

Given the aggressive nature and generally poor prognosis of MPM, treatment decisions in metastatic settings must consider a holistic assessment of disease burden, functional impairment, life expectancy, and patient quality of life (QoL). Distant metastasis significantly influences patients' eligibility for various treatments, including surgery. Although palliative care is often prioritized for metastatic MPM due to limited curative options, accurate diagnosis remains crucial to avoid unnecessary, aggressive procedures and preserve QoL. Chemotherapy, particularly pemetrexed plus platinum, remains the established standard first‐line option for unresectable MPM, though achieving the desired effect is challenging [32]. To address this limitation, anti‐angiogenic agents and immunotherapies have been explored as alternative strategies. Notably, the National Comprehensive Cancer Network (NCCN) guidelines have approved bevacizumab, a monoclonal anti‐VEGF antibody, in combination with first‐line platinum‐based chemotherapy based on promising results from phase 3 clinical trials [33]. While the clinical benefit of such regimens or others in patients with distant metastasis remains inadequately characterized, subgroup analyses are warranted to assess their efficacy in this high‐risk population.

Our analysis underscores the importance of recognizing metastatic spread patterns in MPM. Brain metastases, while relatively common in systemic cancers affecting 20%–40% of adults [34], are less frequent in MPM. Previous reports indicated a 5.3% incidence [25], whereas our analysis revealed an incidence of only 1.6%, with a dismal median overall survival of approximately 2 months. Despite the small sample size, incidental detection of brain metastases in asymptomatic patients highlights the need for proactive brain imaging in high‐risk individuals, particularly those under 65 years of age or with stage IV disease, even in the absence of neurologic signs [25]. Given the substantial impact on QoL, timely detection and supportive care for brain metastases are imperative. In late‐stage MPM, the lung emerges as the most common site of metastasis (18.96%), specifically involving the contralateral lung, distinct from local ipsilateral spread. Previous studies reported that lymph node metastasis is a negative factor affecting cancer prognosis. We found that patients with lymph node metastases have relatively better survival than those with metastases at other sites, possibly owing to the fact that lymph nodes are downstream drainage regions of tumors, and their colonization is presumed to be an intermediary step in dissemination to distant sites [19]. This suggests that when the primary tumor is well‐controlled and metastasis is limited to regional lymph nodes, applying local treatment strategies—such as minimally invasive surgery, radiotherapy, or radiofrequency ablation, as practiced in other tumor types—may offer greater benefits for patients with MPM. While comparative survival analysis across metastatic sites—such as the contralateral lung, bone, liver, and brain—did not yield statistically significant differences, this may stem from the limited detection sensitivity of conventional imaging modalities during early metastatic stages.

Despite offering novel insights, this study has limitations inherent to the SEER database. First, the SEER database lacks detailed information on metastases to organs beyond bone, brain, liver, and lung, restricting a comprehensive analysis of survival impacts across all metastatic sites. Second, missing clinical data in SEER limited the inclusion of all diagnosed MPM cases, and our exclusion of patients without pathological confirmation may introduce selection bias. Third, the absence of crucial clinical variables such as asbestos exposure, primary tumor site, and smoking in the SEER data may influence the accurate assessment of other variables' effects on outcomes. Lastly, the timing of metastasis development (pre‐ or post‐surgery) is not captured, impeding assessment of temporal patterns and identification of dynamic risk factors for metastatic progression.

## 5 Conclusion

This study highlights the critical impact of distant metastasis as an independent prognostic factor in MPM, with substantial influence on overall survival. Patients with solitary distant lymph node metastases demonstrated relatively favorable outcomes, whereas the presence of multiple metastatic sites was associated with poorer prognosis. Additionally, histologic subtype, T‐stage, N‐stage, and tumor laterality were identified as key risk factors for the development of distant metastases. Building upon these findings, we developed a robust prognostic model capable of individualized survival probabilities. Our large‐scale, population‐based analysis emphasizes the clinical relevance of site‐specific metastatic patterns and their prognostic implications. This work supports the implementation of enhanced screening protocols for distant metastases and encourages personalized treatment strategies. Ultimately, our findings provide valuable insights for improving the clinical management and decision‐making process in MPM care.

## Author Contributions

J.Y. and K.D.Y. contributed to conceptualization, methodology design, visualization, writing of the original draft, and supervision of the study. C.P. was responsible for data collection and curation. B.R.G. conducted data validation. Q.W.Y., C.H., and Q.W. took charge of formal analysis. K.D.Y. and Y.B.L. performed the investigation and project administration, and conducted writing review and editing. All authors confirmed the underlying data and reviewed and approved the final manuscript.

## Ethics Statement

The authors have nothing to report.

## Conflicts of Interest

The authors declare no conflicts of interest.

## Supporting information


**Figure S1:** Standardized percent of bias across covariates in selected clinical variables before (denoted in dot) and after (denoted in cross) Propensity Score Matching (PSM). N‐stage, Lymph node stage; T‐stage, Tumor stage.
**Figure S2:** Kaplan–Meier survival curves for MPM patients with DM (distant metastasis) and NDM (non‐distant metastasis) in total PSM samples group by histology **(A)**, surgery **(B)**, chemotherapy **(C)**, N‐stage **(D)**, and age **(E)**. ***, *p* < 0.01; **, *p* < 0.01; *, *p* < 0.05; Comparisons of insignificance were not marked.
**Figure S3:** Forest plots illustrating the results of multivariable Cox regression analyses stratified by histologic subtypes.
**Table S1:** Patterns of distant metastases for MPM patients.

## Data Availability

The data that support the findings of this study are available from the corresponding author upon request.
