# Supplementary material for: Distant Metastatic Pattern and Its Prognostic Significance in Malignant Pleural Mesothelioma: A Population‐Based Study Based on a Machine Learning Model
Source: Clin Respir J. 2025 Nov 11;19(11):e70133. doi: 10.1111/crj.70133 (PMC12603624; doi:10.1111/crj.70133)
Supplement: Supplementary file 1 — Figure S1: Standardized percent of bias across covariates in selected clinical variables before (denoted in dot) and after (denoted in cross) Propensity Score Matching (PSM). N‐stage, Lymph node stage; T‐stage, Tumor stage. Figure S2: Kaplan–Meier survival curves for MPM patients with DM (distant metastasis) and NDM (non‐distant metastasis) in total PSM samples group by histology (A), surgery (B), chemotherapy (C), N‐stage (D), and age (E). ***, p < 0.01; **, p < 0.01; *, p < 0.05; Comparisons of insignificance were not marked. Figure S3: Forest plots illustrating the results of multivariable Cox regression analyses stratified by histologic subtypes. Table S1: Patterns of distant metastases for MPM patients. [file CRJ-19-e70133-s001.docx]

**Supplementary Figures**

**
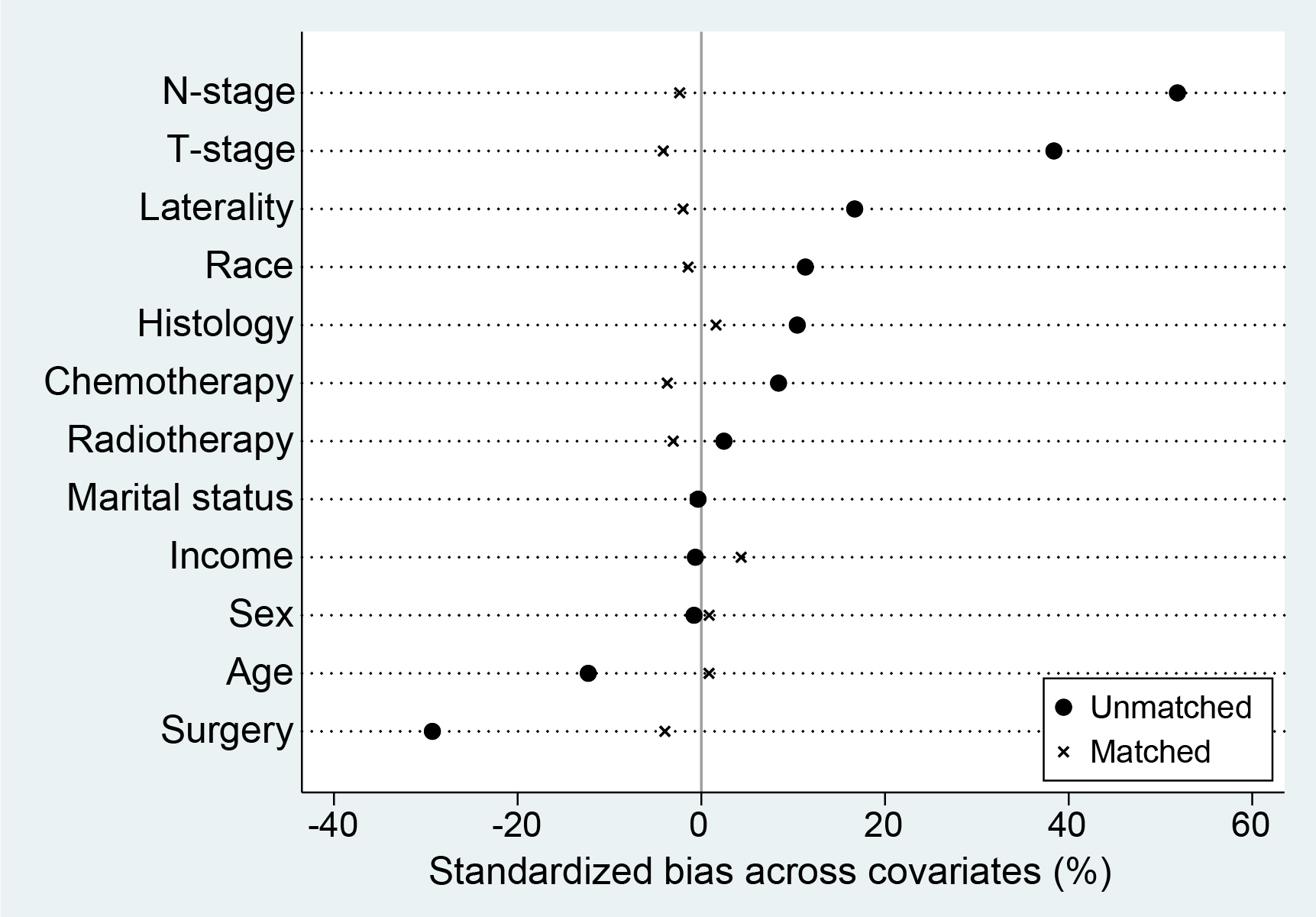
**

**Supplementary Figure 1.** Standardized percent of bias across covariates in selected clinical variables before (denoted in dot) and after (denoted in cross) Propensity Score Matching (PSM). N-stage, Lymph node-stage; T-stage, Tumor-stage.


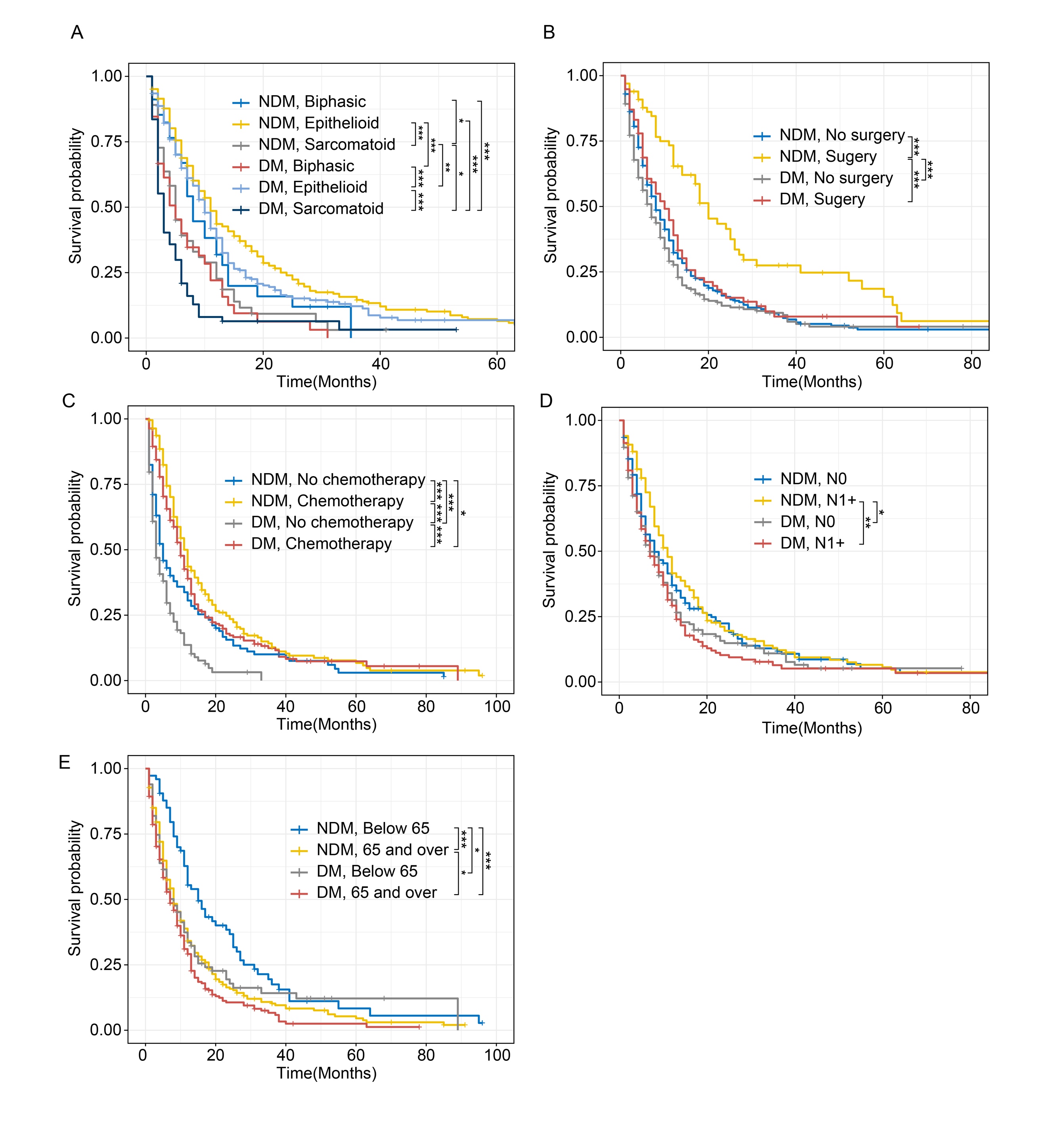


**Supplementary Figure 2.** Kaplan-Meier survival curves for MPM patients with DM (distant metastasis) and NDM (non-distant metastasis) in total PSM samples group by histology **(A)**, surgery **(B)**, chemotherapy **(C)**, N-stage **(D)**, and age **(E)**. ***, *p* < 0.01; **, *p* < 0.01; *, *p* < 0.05; Comparisons of insignificance were not marked.

**
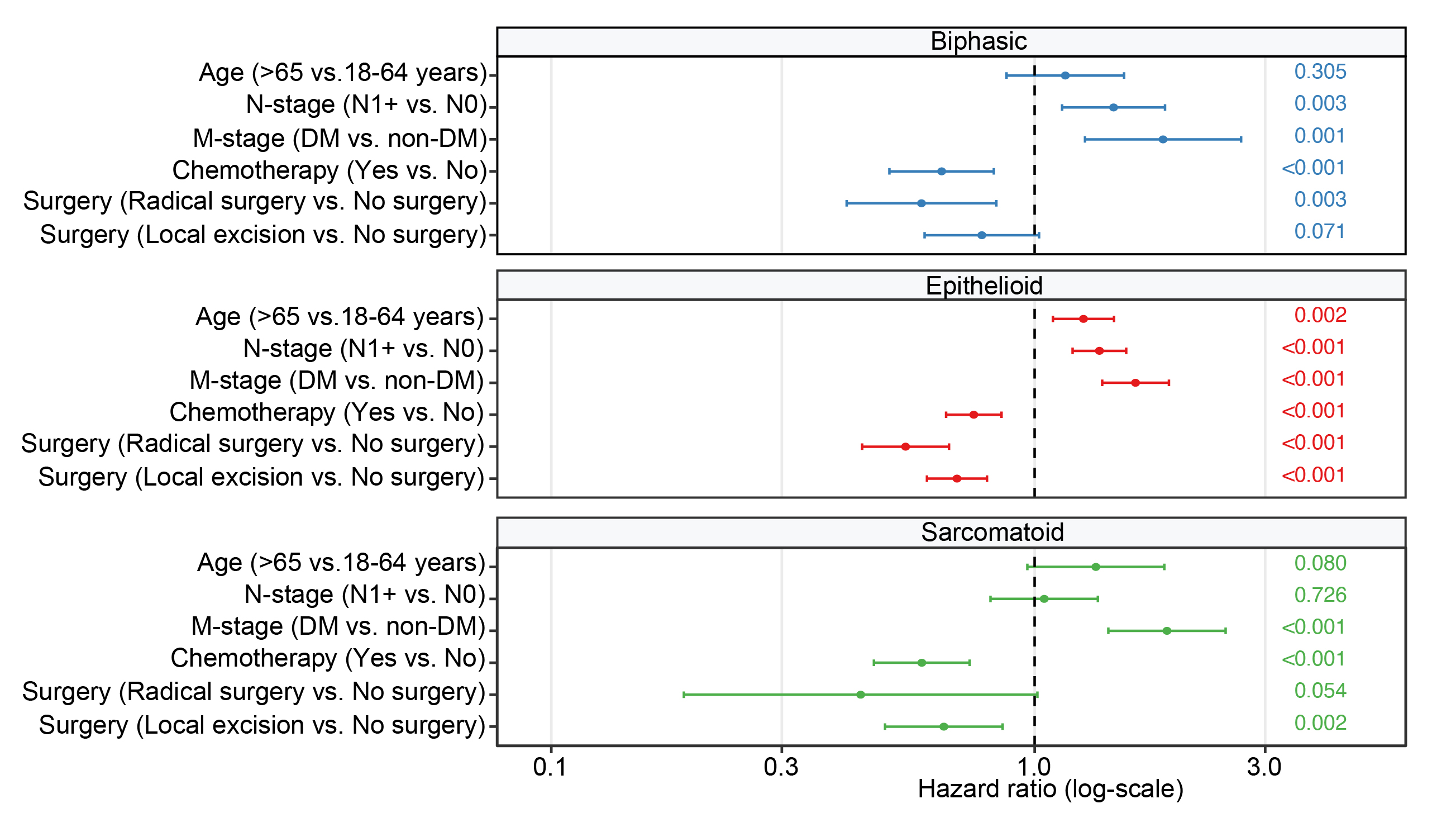
**

**Supplementary Figure 3.** Forest plots illustrating the results of multivariable Cox regression analyses stratified by histologic subtypes.

**Supplementary Table1.** Patterns of distant metastases for MPM patients.

Abbreviation: NOS, Not otherwise specific.
